# Diagnostic potential of multimodal neuroimaging in posttraumatic stress disorder

**DOI:** 10.1371/journal.pone.0177847

**Published:** 2017-05-30

**Authors:** Jooyeon Jamie Im, Binna Kim, Jaeuk Hwang, Jieun E. Kim, Jung Yoon Kim, Sandy Jeong Rhie, Eun Namgung, Ilhyang Kang, Sohyeon Moon, In Kyoon Lyoo, Chang-hyun Park, Sujung Yoon

**Affiliations:** 1 Ewha Brain Institute, Ewha Womans University, Seoul, South Korea; 2 Interdisciplinary Program in Neurosciences, College of Natural Sciences, Seoul National University, Seoul, South Korea; 3 Department of Psychiatry, Soonchunhyang University College of Medicine, Seoul, South Korea; 4 Department of Brain and Cognitive Sciences, Ewha Womans University, Seoul, South Korea; 5 College of Pharmacy and Division of Life and Pharmaceutical Sciences, Ewha Womans University, Seoul, South Korea; 6 Graduate School of Pharmaceutical Sciences, Ewha Womans University, Seoul, South Korea; Yale University, UNITED STATES

## Abstract

Despite accumulating evidence of physiological abnormalities related to posttraumatic stress disorder (PTSD), the current diagnostic criteria for PTSD still rely on clinical interviews. In this study, we investigated the diagnostic potential of multimodal neuroimaging for identifying posttraumatic symptom trajectory after trauma exposure. Thirty trauma-exposed individuals and 29 trauma-unexposed healthy individuals were followed up over a 5-year period. Three waves of assessments using multimodal neuroimaging, including structural magnetic resonance imaging (MRI) and diffusion-weighted MRI, were performed. Based on previous findings that the structural features of the fear circuitry-related brain regions may dynamically change during recovery from the trauma, we employed a machine learning approach to determine whether local, connectivity, and network features of brain regions of the fear circuitry including the amygdala, orbitofrontal and ventromedial prefrontal cortex (OMPFC), hippocampus, insula, and thalamus could distinguish trauma-exposed individuals from trauma-unexposed individuals at each recovery stage. Significant improvement in PTSD symptoms was observed in 23%, 52%, and 88% of trauma-exposed individuals at 1.43, 2.68, and 3.91 years after the trauma, respectively. The structural features of the amygdala were found as major classifiers for discriminating trauma-exposed individuals from trauma-unexposed individuals at 1.43 years after the trauma, but these features were nearly normalized at later phases when most of the trauma-exposed individuals showed clinical improvement in PTSD symptoms. Additionally, the structural features of the OMPFC showed consistent predictive values throughout the recovery period. In conclusion, the current study provides a promising step forward in the development of a clinically applicable predictive model for diagnosing PTSD and predicting recovery from PTSD.

## Introduction

Posttraumatic stress disorder (PTSD) represents a characteristic constellation of symptoms related to uncontrollable state of fear following the experience of a traumatic event [1]. Due to the direct relevance of PTSD symptoms to fear response, brain regions of the fear circuit have important roles in the pathogenesis of PTSD [2]. The amygdala, as a critical brain region mainly involved in fear learning and expression, is central to the fear circuit [3, 4], and subcortical regions with structural connections to the amygdala such as the hippocampus, insula, and thalamus may play a part in the fear circuit [5]. In addition, it has been reported that the prefrontal cortex is specifically involved in the pathophysiology of PTSD in relation to other anxiety disorders [6].

In PTSD, neuroanatomical abnormalities involving brain regions of the fear circuit have been observed in terms of changes in connectivity features as well as local features in both function and structure [7, 8]. Despite increasing knowledge about such brain changes that are involved in the pathophysiology of PTSD, the potential use of brain features for diagnosing PTSD or predicting symptom changes during recovery has rarely been investigated.

In our previous studies on the survivors of a subway fire disaster, we found that increased cortical thickness in the dorsolateral prefrontal cortex [9] and elevated structural connectivity between the amygdala and orbitofrontal and ventromedial prefrontal cortex (OMPFC) [10] were related to PTSD symptom improvement, and such involvement of specific brain structural features could dynamically change during recovery from PTSD. These findings seem to provide important implications for the use of potential neuroanatomical markers in diagnosing PTSD and predicting recovery from PTSD. For instance, various brain structural features from multimodal neuroimaging, rather than a univariate feature, may need to be considered as potential classifiers for robust diagnosis of PTSD. In addition, time-dependent variability in predictive values of brain structural features may also be taken into account, considering the dynamic nature of neuroanatomical changes during the development of and recovery from PTSD.

With respect to applying neuroimaging for disease diagnosis, recent advances in machine learning methods for classification or clustering may provide opportunities to identify predictive models based on a particular subset of neurobiological markers from neuroimaging data [11]. As compared to the conventional analysis for comparing groups, machine learning approaches are likely to be purely discriminative methods, and multivariate brain features in particular from multimodal neuroimaging can be fully used to better classify or predict the disease status in the framework of machine learning [12, 13]. Although machine learning approaches incorporating neuroimaging data have been employed to predict various psychiatric diseases [14], there have been few attempts to characterize specific sets of potential neuroanatomical markers for classifying or predicting the status of trauma exposure, particularly in the time course of recovery.

In this study, a longitudinal multimodal neuroimaging data of disaster survivors were used to identify a specific subset of neuroanatomical features, which can best characterize the trauma-exposed group, at each stage of recovery from PTSD. Brain regions in the amygdala-centered fear circuit including the OMPFC, hippocampus, insula, and thalamus were considered as regions of interest (ROIs). A total of 23 quantitative local, connectivity, and network features were extracted from structural magnetic resonance imaging (sMRI) and diffusion-weighted MRI (dMRI) datasets. By employing a classification approach incorporating multimodal characteristics of the brain regions in the fear circuitry, tentative neuroanatomical features that may differentiate trauma-exposed group from trauma-unexposed group were identified and their predictive values were estimated during the 5 years of follow up period.

## Methods

### Participants

The trauma-exposed group in this study included survivors of a subway fire disaster in South Korea [9, 10]. The initial assessment was conducted for 38 individuals aged 18 to 50 years at approximately 1.65±0.74 months after the disaster (time 0). Among them, 30 completed the time 1 assessment (time 1), 25 completed up to the time 2 assessment (time 2), and 17 completed up to the time 3 assessment (time 3). The diagnosis and symptoms severity of PTSD were assessed using the Structural Clinical Interview for the Diagnostic and Statistical Manual of Mental Disorders, Fourth Edition (DSM-IV) [15] and Clinician-Administered Posttraumatic Stress Disorder Scale (CAPS) [16], respectively. Details on exclusion criteria for participation in this study are described in S1 Text. Age- and sex-matched healthy controls were initially enrolled as the trauma-unexposed group. Out of 36 healthy individuals from the original cohort [9], 29 age- and sex-matched healthy individuals, who completed two or more assessments, were included as the trauma-unexposed group in the present study [10] (Supplementary Table). Among them, all 29 completed up to the time 2 assessment and 21 completed up to the time 3 assessment.

Serial assessment data of 30 trauma-exposed individuals and 29 trauma-unexposed individuals who undertook two or more assessments were eventually included in the final analysis.

Both the trauma-exposed and unexposed groups underwent three waves of assessments including multimodal neuroimaging and comprehensive psychiatric evaluations over a 5-year period at approximately 1.3-year intervals: time 1 at 1.43±0.22 years, time 2 at 2.68±0.32 years, and time 3 at 3.91±0.33 years after the trauma. Between the trauma-exposed and unexposed groups, there were no differences in sex, age, education, and handedness (Table 1A).

**Table 1:** Demographic and clinical characteristics of participants.

(A) Comparison of demographic characteristics between trauma-exposed and unexposed individuals			
	**Trauma-exposed** **group** (n = 30)	**Trauma-unexposed** **group** (n = 29)	**Statistical significance**
Sex (male:female)	11:19	11:18	NS
Age (mean±SD)	27.0±8.8 years	26.4±6.3 years	NS
Education (mean±SD)	13.6±2.2 years	13.9±2.0 years	NS
Handedness (right:left)	28:2	28:1	NS
(B) Changes in clinical characteristics in trauma-exposed individuals			
**Stage of recovery from PTSD**		**CAPS total score** (mean±SD)	**# of individuals diagnosed as PTSD**
time 0		87.1±12.6	30/30 (100%)
time 1		54.0±14.1	23/30 (77%)
time 2		45.6±11.8	12/25 (48%)
time 3		35.1±15.0	2/17 (12%)

SD, standard deviation; NS, not significant at P = 0.05; PTSD, posttraumatic stress disorder; CAPS, Clinician-Administered Posttraumatic Stress Disorder Scale.

Written informed consent was obtained from all participants. The study protocol was approved by the Institutional Review Board of the Seoul National University Hospital and all procedures contributing to this work comply with the latest version of the Declaration of Helsinki.

### Neuroimaging data acquisition

Serial brain scans were performed using a Signa EXCITE 3.0T MRI system (GE Healthcare, Milwaukee, WI, USA). At each assessment, high-resolution T1-weighted sMRI data were obtained using a three-dimensional inversion recovery spoiled gradient-echo pulse sequence with the following parameters: echo time (TE) = 1.4 ms, repetition time (TR) = 5.7 ms, flip angle (FA) = 20°, matrix size = 256 x 256, field of view (FOV) = 22 cm, and slice thickness = 0.7 mm. Also, dMRI data were acquired using a dual spin-echo echo-planar imaging sequence with the following parameters: 25 diffusion directions, b values = 0 s/m^2^ for no diffusion weighting and 1,000 s/m^2^ for diffusion weighting, TE = 90 ms, TR = 10,000 ms, matrix = 256 x 256, FOV = 24 cm, and slice thickness = 3.5 mm.

### Regions of interest definition

Brain regions of the fear circuitry including the amygdala, OMPFC, hippocampus, insula, and thalamus were selected as ROIs for the extraction of brain structural features. The OMPFC was determined to consist of the ventromedial and adjacent orbitofrontal cortices [17, 18]. Detailed methods regarding the delineation and subsequent processing of ROI masks are described in S1 Text.

### Structural features extraction

**Fig 1:**
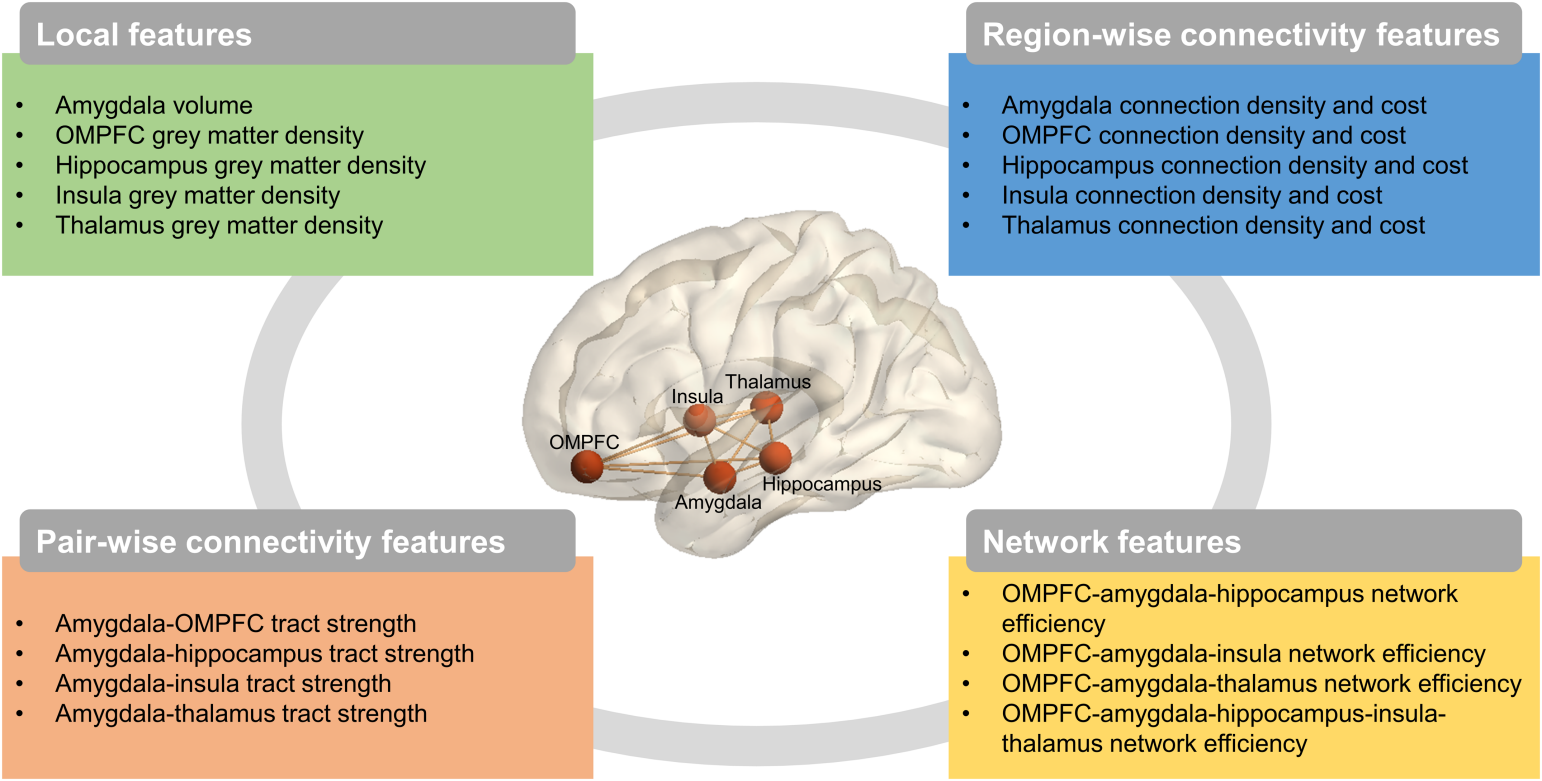
Multimodal characteristics of the amygdala, OMPFC, hippocampus, insula, and thalamus assessed at each time point. A set of candidate brain structural features was derived from multimodal neuroimaging data analysis, which comprehensively characterized local, region-wise connectivity, pair-wise connectivity, and network features of the amygdala, orbitofrontal and ventromedial prefrontal cortex (OMPFC), hippocampus, insula, and thalamus.

A total of 23 brain structural features were derived from the analysis of multimodal neuroimaging data including sMRI and dMRI data (Fig 1 and Supplementary Fig). For the five ROIs, structural features included four categories: (i) local features from sMRI data including the volume of the amygdala and the grey matter density of the other four ROIs; (ii) region-wise connectivity features from dMRI data including connection density and connection cost in each ROI; (iii) pair-wise connectivity features from dMRI data corresponding to tract strength between the amygdala and each of the other four ROIs; and (iv) network features from dMRI data including network efficiency for the network composed of all five ROIs and for each subnetwork composed of three ROIs.

#### Local features

The volume of the amygdala was measured by manual segmentation, and the grey matter density of the OMPFC, hippocampus, insula, and thalamus was assessed by employing voxel-based morphometry (For details, refer to S1 Text). Grey matter density of each ROI was provided by averaging values over all voxels in the ROI.

#### Region-wise connectivity features

Fiber tracts among the five ROIs were reconstructed using the streamlining method as implemented in the TrackVis software (http://trackvis.org/) (For details, refer to S1 Text). For each pair of the ROIs, reconstructed fiber tracts were counted, and then the number of fiber tracts was divided by the summed volume of interconnected ROIs to measure connection density [19], and it was multiplied by the average length of fiber tracts to compute connection cost [20]. Region-wise connection density and cost were obtained by averaging the pair-wise values for each ROI.

#### Pair-wise connectivity features

Fiber tracts between the amygdala and each of the other four ROIs were reconstructed using the probabilistic method as implemented in the FDT tool of the FSL software (http://fsl.fmrib.ox.ac.uk/) (For details, refer to S1 Text). The seed-based classification procedure [21] was adopted to assess relative tract strength between the amygdala and the other four ROIs. For each voxel of the amygdala, the count ratio of fiber tracts reaching only the specific ROI to fiber tracts reaching any of the four ROIs [22, 23] was computed. Relative tract strength was provided for each pair of the ROIs (amygdala-OMPFC, amygdala-hippocampus, amygdala-insula, and amygdala-thalamus) by averaging the ratio values over all voxels except those with the ratio value below 0.01.

#### Network features

*Network efficiency* measures how efficiently information is exchanged over a network [24]. The measure was computed for a structural network that was constructed based on the density of reconstructed fiber tracts among five bilateral ROIs (OMPFC-amygdala-hippocampus-insula-thalamus), and each of their subnetworks determined for three bilateral ROIs (OMPFC-amygdala-hippocampus, OMPFC-amygdala-insula, and OMPFC-amygdala-thalamus).

Local feature values and region-wise and pair-wise connectivity feature values were summed or averaged over the two hemispheres to preclude preference for either hemisphere. In addition, all feature values were adjusted for age and sex, and amygdala volume was additionally adjusted for the intracranial volume. For each structural feature, the trauma-exposed individuals' values were converted into standardized *Z*-scores in terms of the standard deviation (SD) from the mean of the trauma-unexposed group. Resultantly, the feature values of the trauma-exposed group had a distribution with reference to the mean and SD values of the trauma-unexposed group, such that a negative *Z*-score of a trauma-exposed individual indicated that their adjusted feature value was below the mean of the trauma-unexposed group in SD units.

### Classification model search

**Fig 2:**
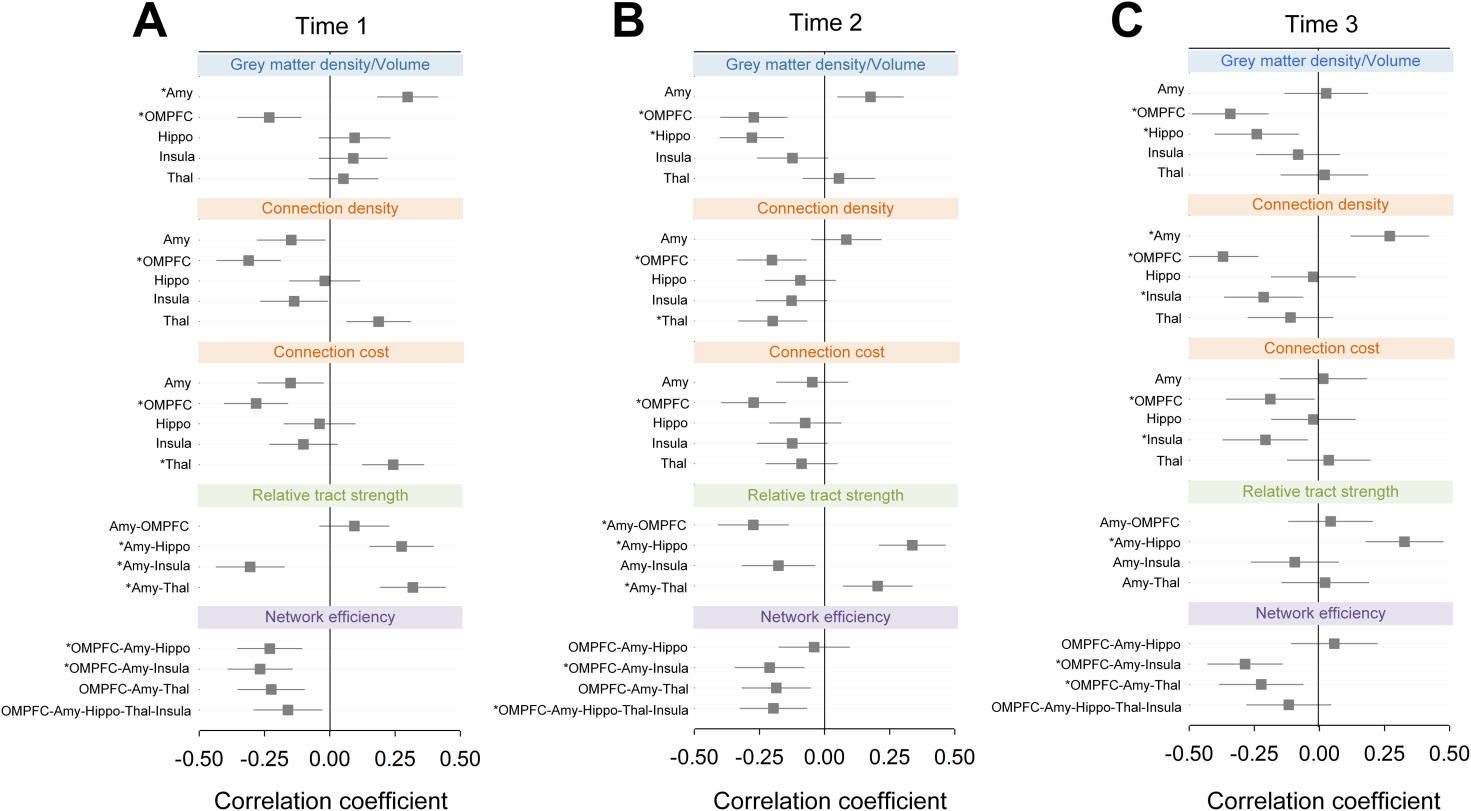
The relationships between candidate brain structural features and the group membership at each time point. The graph presents point-biserial correlation coefficients (*r*) between candidate features and the group membership at (A) time 1, (B) time 2, and (C) time 3 assessments. Error bars represent standard errors, which were calculated using 5,000 bootstraps. Asterisks in each graph indicate the first 10 brain structural features based on the rank of the absolute *r* values. Amy, amygdala; OMPFC, orbitofrontal and ventromedial prefrontal cortex; Hippo, hippocampus; Thal, thalamus.

Classification between the trauma-exposed and unexposed groups was performed based on the 23 multimodal features (Fig 1 and Supplementary Fig) of the five ROIs. We used logistic regression with ridge estimators as a classification algorithm [25] as implemented in the Waikato Environment for Knowledge Analysis interface [26]. At each time point, seven different classification models with 4 to 10 brain structural features were constructed. The priority of the brain structural features among 23 was determined based on the rank of point-biserial correlation coefficients of individual features (Fig 2).

For each classification model, performance for correctly assigning an individual to the trauma-exposed group was assessed in terms of the area under a receiver operating characteristic curve (AUC). We used bootstrap tests (n = 1,000) to assess whether an AUC is significantly greater than the chance level (AUC = 0.5) [27] and to compare AUCs between any two classification models [28].

The classification model with the greatest AUC was then determined as the best model for classifying the trauma-exposed group from the trauma-unexposed group at each time point. Having identified the subset of brain structural features corresponding to the best classification model, the accuracy of each individual feature on its own was measured using the AUC. All statistical analysis was performed using the Stata/SE software (release 13.1) (Stata Corp LP, College Station, TX, USA), and the statistical significance level was set at *P* = 0.05 in all statistical inferences.

## Results

### PTSD symptoms improvement

Most of trauma-exposed individuals showed significant improvement in PTSD symptoms, as measured with the CAPS, over time. Among all trauma-exposed individuals who were diagnosed with PTSD at time 0, 7 of 30 (23%) at time 1, 13 of 25 (52%) at time 2, and 15 of 17 (88%) at time 3 improved in such a way that their CAPS scores did not meet the diagnostic criteria for PTSD (Table 1B).

### Brain structural features for group classification at time 1

**Fig 3:**
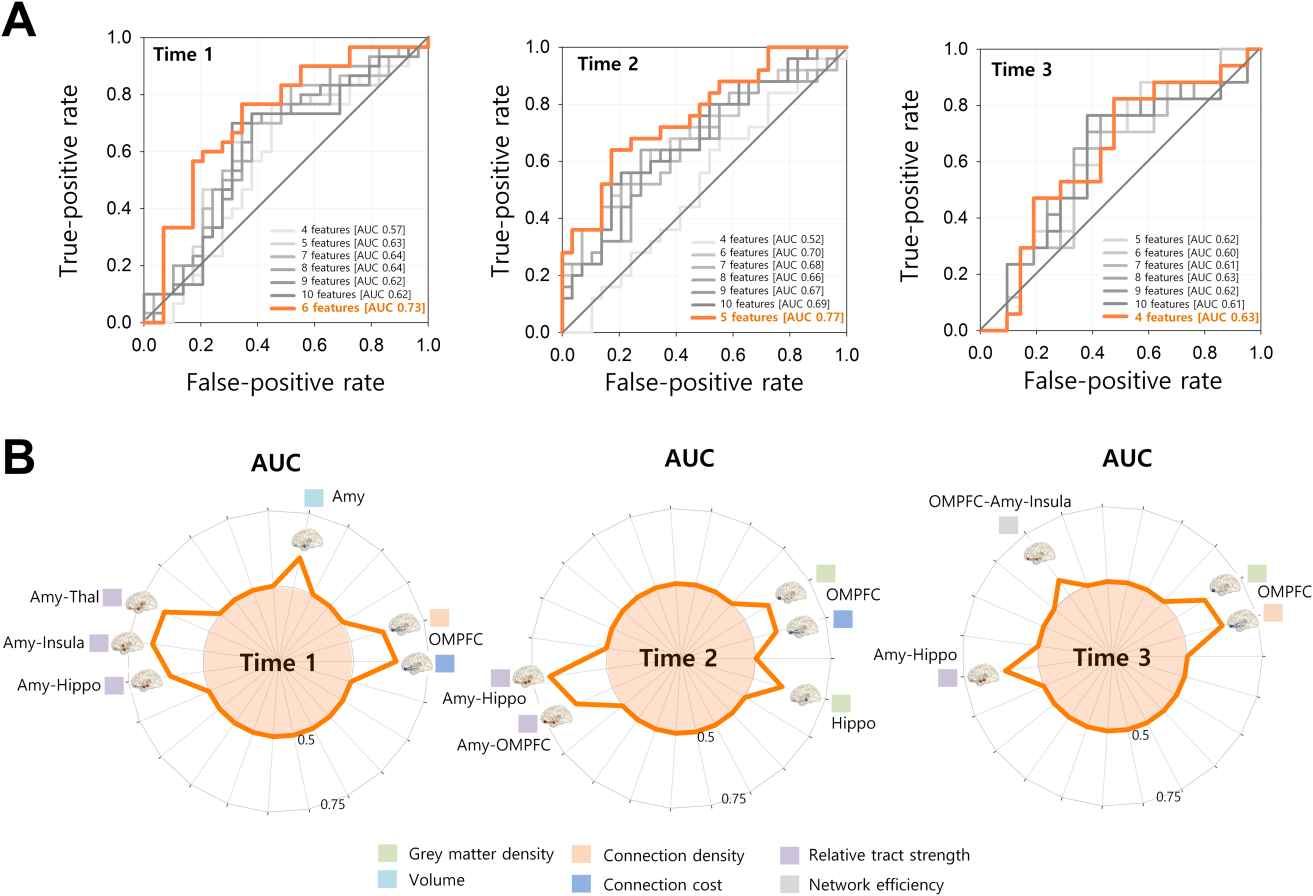
Multimodal brain structural features and their contribution to the classification of the trauma-exposed group from the trauma-unexposed group at each time point. (A) Receiver operating characteristic curves of classification models at each time point are presented. Performance of each model for classifying the trauma-exposed group from the trauma-unexposed group as a function of the subset of candidate features was measured using the AUC. The model showing the best classification performance at each time point is plotted in orange color. (B) The best subset of multimodal features for classifying the trauma-exposed group from the trauma-unexposed group at each time point is presented. Classification performance measured using the AUC for individual features is plotted in radar graphs. AUC, area under a receiver operating characteristic curve; Amy, amygdala; OMPFC, orbitofrontal and ventromedial prefrontal cortex; Hippo, hippocampus; Thal, thalamus.

The classification model with six brain structural features successfully distinguished the trauma-exposed group from the trauma-unexposed group at time 1 with the largest AUC above the chance level (AUC = 0.73, 95% CI = 0.54 to 0.91, *P* = 0.01) (Fig 3A). The second best classification model with seven brain structural features did not reach statistical significance (AUC = 0.64, 95% CI = 0.47 to 0.82, *P* = 0.06). Brain structural features for the best classification model included the amygdala-insula tract strength, amygdala-thalamus tract strength, OMPFC connection density, OMPFC connection cost, amygdala-hippocampus tract strength, and amygdala volume in the decreasing order of their individual performance (Fig 3B and Table 2).

**Table 2:** Classification accuracy of individual brain structural features included in the best model for distinguishing trauma-exposed individuals from trauma-unexposed individuals at time 1, 2, and 3.

Brain structural feature	AUC	SEM
**Time 1**		
Amygdala-insula tract strength	0.67	0.13
Amygdala-thalamus tract strength	0.66	0.11
OMPFC connection density	0.64	0.13
OMPFC connection cost	0.61	0.14
Amygdala-hippocampus tract strength	0.61	0.13
Amygdala volume	0.60	0.13
**Time 2**		
Amygdala-hippocampus tract strength	0.69	0.11
Amygdala-OMPFC tract strength	0.63	0.16
Hippocampus grey matter density	0.60	0.15
OMPFC grey matter density	0.59	0.15
OMPFC connection cost	0.58	0.15
**Time 3**		
OMPFC connection density	0.63	0.15
OMPFC grey matter density	0.61	0.18
Amygdala-hippocampus tract strength	0.61	0.18
OMPFC-amygdala-insula network efficiency	0.56	0.19

AUC, area under the receiver operating characteristic curve; SEM, standard error of mean; OMPFC, orbitofrontal and ventromedial prefrontal cortex.

### Brain structural features for group classification at time 2

The classification model including five brain structural features showed the best performance for classifying the trauma-exposed group from the trauma-unexposed group at time 2 with the largest AUC above the chance level (AUC = 0.77, 95% CI = 0.56 to 0.97, *P* = 0.005) (Fig 3A). The second best classification model with six brain structural features exhibited statistical significance (AUC = 0.70, 95% CI = 0.50 to 0.90, *P* = 0.03) as well, but its performance was inferior to that of the best classification model with a statistically significant difference in the AUC (ΔAUC = 0.07, *P* = 0.04). Brain structural features that contributed to the best classification model were amygdala-hippocampus tract strength, amygdala-OMPFC tract strength, hippocampus grey matter density, OMPFC grey matter density, and OMPFC connection cost in the decreasing order of their individual performance (Fig 3B and Table 2).

### Brain structural features for group classification at time 3

The best classification model with the largest AUC (AUC = 0.63, 95% CI = 0.37 to 0.89) (Fig 3A) included four brain structural features, but it failed to classify the trauma-exposed group from the trauma-unexposed group with statistical significance above the chance level (*P* = 0.16). Brain structural features for the best classification model included OMPFC connection density, OMPFC grey matter density, amygdala-hippocampus tract strength, and OMPFC-amygdala-insula network efficiency in the deceasing order of their individual performance (Fig 3B and Table 2).

## Discussion

A unified model integrating multiple variables via machine learning methods may allow the application of multimodal neuroimaging data for clinical diagnosis of neuropsychiatric disorders. To the best of our knowledge, this is the first study to provide a preliminary evidence for the use of multimodal neuroimaging features as potential classifiers of trauma-related symptom phenotypes. The present study included longitudinal multimodal neuroimaging data acquired from the cohort of disaster survivors who were diagnosed with PTSD in the early aftermath of the trauma. This study design enabled us to assess dynamic changes in predictive values of multimodal brain structural features in the time course of recovery from PTSD. We demonstrated that brain structural features extracted from multimodal neuroimaging data could be effectively applied to distinguishing the trauma-exposed group from the trauma-unexposed group at different phases of the development and improvement of PTSD symptoms, which highlighted our proposed concerns about diagnostic potential for PTSD.

The best classification models in predicting individuals with trauma exposure required 6 brain structural features at time 1, 5 brain structural features at time 2, and 4 brain structural features at time 3. Of note, performance of the best classification model did not reach statistical significance at the recovery phase as late as time 3, revealing that PTSD-induced changes in the amygdala-centered fear circuit were likely to normalize as PTSD symptoms substantially improved. In addition to the decreasing trend in the number of brain structural features included in the best classification models at time 3, the contributing brain structural features were partly overlapped along the time course. These findings indicate that the predictive values of brain structural features dynamically change over time, suggesting preserved or changing roles of brain structural features across the trajectory of the development and improvement of PTSD symptoms.

A set of brain structural features that mainly contributed to the classification model at each time is likely to indicate contrasting aspects of the brains between the trauma-exposed and unexposed groups. In particular, such time-dependent contributions of brain structural features may have implications regarding whether the plastic changes during the recovery process might reflect the reinstatement and restoration or compensation and adaptation of the brain [29, 30].

At time 1, when most individuals had a high level of PTSD symptoms, prominent brain structural characteristics that distinguished the trauma-exposed group from the trauma-unexposed group included amygdala volume as a local feature, OMPFC connection density and cost as region-wise connectivity features, and amygdala-insula, amygdala-thalamus, and amygdala-hippocampus tract strength as pair-wise connectivity features. It should be noted that the second best classification model at time 1 had also a trend of statistical significance (*P* = 0.06). As compared with the best classification model, this model additionally included a brain structural feature of OMPFC-amygdala-insula network efficiency implying its potential role in classifying the groups at 1.43 years after the trauma.

Amygdala volume was not included in the best classification model at times 2 and 3. This finding potentially implies that amygdala volume-related pathology may be reversible with PTSD symptom improvement. In addition, among pair-wise connectivity features that had predictive values at time 1, amygdala-insula and amygdala-thalamus tract strengths were not included in the best classification model at times 2 and 3 partly due to the normalization of these features, whereas amygdala-hippocampus tract strength remained abnormal even at the time point as late as time 3. However, these findings should be interpreted with caution in that the best predictive model for classifying the diagnosis group can be determined in relation to the combination of several predictors, rather than its sole statistical significance.

Involvement of OMPFC connection density and cost in distinguishing the trauma-exposed group from the trauma-unexposed group at time 1 and their persistent predictive values until times 2 and 3 seem to be characteristic brain changes in the development and improvement of PTSD. Moreover, OMPFC grey matter density had predictive values concurrently from time 2 onward. The prefrontal cortex, in addition to limbic structures such as the amygdala, has been suggested to play a key role in the distributed network of brain regions involved in fear behavior [31, 32]. Indeed, decreased activation in the OMPFC has been observed during recollection of traumatic events in individuals with PTSD [33–35], and furthermore, such altered activation in the OMPFC was shown to be negatively associated with PTSD symptoms severity [36], supporting the hypothesis that impairment of fear extinction in PTSD patients could be related to a failure of OMPFC activation [37]. The augmentation of OMPFC grey matter density as well as connectivity density and cost during the recovery process may suggest the necessity of the reinforced role of the OMPFC in PTSD symptom improvement. This is in line with our previous study that showed the contribution of increased amygdala-OMPFC tract strength to PTSD symptoms improvement [10].

Taken together, whereas predictive values of amygdala-related local and connectivity features, except amygdala-hippocampus tract strength, disappeared at late phases after normalization, OMPFC-related local and connectivity features consistently had predictive values throughout the recovery period from PTSD. Understanding of the mixed occurrence of restorative and adaptive brain processes in recovery would help to determine recovery phase-dependent predictive models for the diagnosis of PTSD. In this regard, it is noteworthy that brain processes in recovery may be related to preexisting vulnerability factors or trauma-induced abnormalities [38] and the distinction between the two should be considered in constructing predictive models.

The findings of this study highlight the necessity and significance of brain structural features extracted from multimodal neuroimaging data as diagnostic potential for identifying posttraumatic symptom trajectory after trauma exposure. For all three time points, the best classification models included region-wise and pair-wise connectivity features as well as local features. Furthermore, though not statistically significant, the best classification model at time 3 included a network feature. Multimodal features appear to be useful and provide comprehensive information about the brain such that predictive models of better performance could be searched for, and more importantly, they enable more comprehensive understanding about brain processes. For instance, the findings that both local and connectivity features of the amygdala and OMPFC showed time-dependent changes as PTSD symptoms substantially improved may expand a perspective on plasticity of the brain. A search for more accurate and effective predictive models for the diagnosis of PTSD is warranted in future studies employing brain functional and structural imaging data, as brain functional features such as interregional functional coupling and local activation could provide additional predictive values that complement brain structural features.

As mentioned in our previous study [10], one of the limitations is that individuals who had experienced a traumatic event but did not develop PTSD were not included in the study. Thus, the distinction between brain changes following a trauma exposure and those related to the development of PTSD could not be made in the current study. Future studies with trauma-exposed individuals who do not develop PTSD would be necessary to investigate differences in brain trajectories between individuals who develop PTSD and those who do not.

It is noteworthy that a large proportion of the trauma-exposed individuals in the current study has recovered from PTSD at 5 years after the subway disaster. This finding is relatively consistent with the previous longitudinal studies, which provided follow-up assessments for survivors from a single man-made or natural disaster [39–42]. However, the absence of information on the 13 trauma-exposed individuals at time 3 including their PTSD symptom severity and neuroimaging data, due to no longer participating in the study, may be a limitation for the estimation of accurate prevalence rate of PTSD and its recovery trajectory in individuals with an exposure to a single man-made disaster. Moreover, as most of trauma-exposed individuals showed significant improvement in PTSD symptoms, the differences between recovered and unrecovered trauma-exposed individuals could not be distinguished in this study. Such distinctions in the time course of PTSD recovery would provide information for the treatment and prognosis of PTSD, and thus are worth consideration in future studies of larger samples.

In the current study, we proposed the best predictive models based on brain structural features extracted from multimodal neuroimaging at different stages of recovery from PTSD. Although investigating the potential use of multimodal neuroimaging is a promising step forward in the development of neuroanatomy-based diagnosis of PTSD, subsequent validation and extension steps will be needed for clinical application. More complete predictive models would be constructed by including clinical and biological information [43] besides neuroimaging information and even incorporating genetic information [44].

In conclusion, our attempts to distinguish the trauma-exposed group from the trauma-unexposed group using the machine learning approach at different phases of recovery from PTSD allowed us to determine classification models and associated structural features of diagnostic potential for PTSD. The classification models were distinct between the recovery phases, including different subsets of multimodal brain structural features. Understanding that predictive values of brain structural features dynamically change according to the development and improvement phase of PTSD would be needed for successful diagnosis of PTSD.

## Supporting information

S1 Text Supplementary Methods, Supplementary Result, Supplementary Table, and Supplementary Figure. (DOCX)
