# Supplementary material for: Diagnostic potential of multimodal neuroimaging in posttraumatic stress disorder
Source: PLoS One. 2017 May 30;12(5):e0177847. doi: 10.1371/journal.pone.0177847 (PMC5448741; doi:10.1371/journal.pone.0177847)
Supplement: S1 Text — (DOCX) [file pone.0177847.s001.docx]

**Supplementary Methods**

**Participants**

Study participants of this study included survivors of a subway fire disaster that took place on February, 2003 in South Korea. The same cohort has been reported in our previous works [1, 2]. At 1.65±0.74 months after the disaster, the survivors were recruited and the following inclusion and exclusion criteria were applied. Inclusion criteria included a diagnosis of posttraumatic stress disorder (PTSD) by the criteria of Diagnostic and Statistical Manual of Mental Disorders, Fourth Edition (DSM-IV) [3]. Exclusion criteria were the presence of (i) current and lifetime Axis I or major Axis II disorders other than PTSD, (ii) major medical conditions, or (iii) any contraindications to magnetic resonance imaging (MRI). More details about the disaster and inclusion and exclusion criteria are described elsewhere [1, 2].

**Region of interest definition**

**Manual tracing of the amygdala**

As a region of interest (ROI), the amygdala was defined via manual tracing, with reference to its anatomical descriptions [4, 5], using the Analyze software package (Biomedical Imaging Resource, Mayo Foundation, Rochester, MN, USA). Intraclass coefficients on intra-rater and inter-rater rater reliability in the manual tracing were reported in our previous study [1].

***A priori* definition of the OMPFC, hippocampus, insula, and thalamus**

ROIs of the orbitofrontal and ventromedial prefrontal cortex (OMPFC), hippocampus, insula, and thalamus were defined based on the automated anatomical labelling (AAL) map [6] in the Montreal Neurological Institute (MNI) space. Specifically, the OMPFC was determined to comprise ventromedial and adjacent orbitofrontal cortices [7, 8].

**Structural feature extraction: local features**

**Volume of the amygdala**

By counting the number of voxels in the amygdala mask and multiplying the voxel count by voxel volume, the volume of the amygdala was computed. The value of amygdala volume was used in final analysis after adjusting for age, sex, and intracranial volume.

**Grey matter density in the OMPFC, hippocampus, insula, and thalamus**

The mean grey matter density of the ROIs including the OMPFC, hippocampus, insula, and thalamus was estimated using a voxel-based morphometric (VBM) approach [9] following the procedures in the FSL software (http://www.fmrib.ox.ac.uk/fsl). In brief, skull stripping was performed to remove non-brain tissues on T1-weighted images [10] and probabilistic tissue classification of grey matter, white matter, and cerebrospinal fluid was carried out at each voxel [11]. The resultant grey matter images in the structural MRI (sMRI) native space were transformed to the MNI space by affine transformation and averaged to create an initial template. Grey matter images in the sMRI native space were transformed to the initial template again and averaged to create a final study-specific template. Then, each grey matter image in the sMRI native space was finally normalized to the study-specific template. After visual inspection to ensure the quality of registration, the normalized images were smoothed with an isotropic Gaussian Kernel of the FWHM of 4.6 mm.

ROI masks of the OMPFC, hippocampus, insula, and thalamus were resliced to the same dimension as that of normalized grey matter images. The voxel-wise value was averaged over all voxels within each ROI to yield grey matter density [9, 12], which was used in final analysis after adjusting for age and sex.

**Structural feature extraction: region-wise connectivity features**

**Connection density and cost in each ROI**

Diffusion-weighted images were registered to the non-diffusion image (*b* = 0 s/m^2^) by affine transformations in order to correct for head motion and minimize distortions due to eddy current. For the preprocessed diffusion-weighted images, diffusion tensors were calculated on a voxel-by-voxel basis using the Diffusion Toolkit software (http://trackvis.org/dtk). The amygdala mask in the sMRI native space and the masks of the OMPFC, hippocampus, insula, and thalamus in the MNI space were transformed to those in the diffusion-weighted MRI (dMRI) native space. White matter fiber tracts interconnecting each pair of the five ROIs were reconstructed using the Fiber Assignment by Continuous Tracking (FACT) algorithm [13]. For each voxel within individual ROIs, five streamline seeds following the main diffusion direction were selected and initialized. A streamline was stopped when a fiber tract reached a voxel with a fractional anisotropy value lower than 0.1, when it turned an angle greater than 45 degrees or when the streamline exceeded the ROI.

Connection density was calculated as the number of streamlines between each pair of ROIs divided by the volumes of the interconnected ROIs [14]. Connection cost was calculated as the number of streamlines multiplied by their average length [15]. Mean values of connection density and connection cost in each ROI were used in final analysis after adjusting for age and sex.

**Structural feature extraction: pair-wise connectivity features**

**Relative tract strength between the amygdala and each of the other ROIs**

With the amygdala as the seed region and the OMPFC, hippocampus, insula, and thalamus as target regions, probabilistic tractography was performed using the bedpostX tool in the FSL software. Based on probability distributions on fiber directions at each voxel, 5,000 streamline samples starting from each voxel of the amygdala were generated.

For voxels in the amygdala that generated more than 10 streamline samples [16], the ratio of the number of tracts reaching each target region to the number of tracts reaching the four target regions [17, 18] was computed. The voxel-wise value was averaged over all voxels within the amygdala but those with the ratio value below 0.01 to yield relative tract strength between the amygdala and each of the other ROIs (amygdala-OMPFC, amygdala-hippocampus, amygdala-insula, and amygdala-thalamus), which was used in final analysis after adjusting for age and sex.

**Structural feature extraction: network features**

**Network efficiency**

A network is constructed by defining nodes and estimating edges between them. The five bilateral ROIs including the amygdala, OMPFC, hippocampus, insula, and thalamus served as nodes, and a pair of nodes were considered as being structurally connected if a set of streamlines interconnecting them was found. A weight assigned to each edge was determined based on connection density which was defined as the number of streamlines corrected for the sum of volumes of interconnected ROIs.

Network efficiency, defined as the average of inverse shortest length between all pairs of nodes [19], was employed as a network measure of interest. Network efficiency was measured for the connection density-weighted network among the five bilateral ROIs, and each of its subnetworks determined for three bilateral ROIs (OMPFC-amygdala-hippocampus, OMPFC-amygdala-insula, and OMPFC-amygdala-thalamus).

**Supplementary Result**

We performed repeated analyses to determine the best model for classifying the trauma-exposed group from the trauma-unexposed group at times 2 and 3 by replacing missing data with the class mean values of the respective group [20]. Similar results were obtained with these repeated analyses. At time 2, the best classification model with the largest AUC (AUC = 0.78, 95% confidence interval [CI] = 0.58 to 0.98, *P* = 0.002) included five brain structural features (amygdala-hippocampus tract strength, amygdala-OMPFC tract strength, hippocampus grey matter density, OMPFC grey matter density, and OMPFC connection cost). At time 3, the classification models including four brain structural features (AUC = 0.79, 95% confidence interval [CI] = 0.63 to 0.94, *P* < 0.001)(OMPFC connection density, OMPFC grey matter density, amygdala-hippocampus tract strength, and OMPFC-amygdala-insula network efficiency) and five brain structural features (AUC = 0.79, 95% confidence interval [CI] = 0.63 to 0.95, *P* < 0.001)(OMPFC connection density, OMPFC grey matter density, amygdala-hippocampus tract strength, OMPFC-amygdala-insula network efficiency, and amygdala connection density) showed the best performance in classifying the groups.

**Supplementary Table.** Sample size of the trauma-exposed and trauma-unexposed groups in the present study and other published studies of the same cohort.

|  | Trauma-exposed group | | | Trauma-unexposed group | | |
| --- | --- | --- | --- | --- | --- | --- |
|  | Lyoo et al^1^ | Yoon et al^2^ | This study | Lyoo et al^1^ | Yoon et al^2^ | This study |
| time 0 (1.65 m since trauma) | 38 | 38 | 38 | NA | NA | NA |
| time 1 (1.43 y since trauma) | 30 | 30 | 30 | 36 | 29* | 29* |
| time 2 (2.68 y since trauma) | 25 | 25 | 25 | 29 | 29 | 29 |
| time 3 (3.91 y since trauma) | 17 | 17 | 17 | 21 | 21 | 21 |
| * Out of 36 trauma-unexposed individuals of the original cohort [2], 29 age- and sex-matched trauma-unexposed individuals who undertook two or more assessments were included in the final analyses to more accurately characterize a longitudinal trajectory.  ^1^ Lyoo IK, Kim JE, Yoon SJ, Hwang J, Bae S, Kim DJ. The neurobiological role of the dorsolateral prefrontal cortex in recovery from trauma: Longitudinal brain imaging study among survivors of the South Korean subway disaster. Arch Gen Psychiatry. 2011;68(7):701-13.  ^2^ Yoon S, Kim JE, Hwang J, Kang I, Jeon S, Im JJ, et al. Recovery from posttraumatic stress requires dynamic and sequential shifts in amygdalar connectivities. Neuropsychopharmacology. 2017;42(2):454-61. | | | | | | |

**
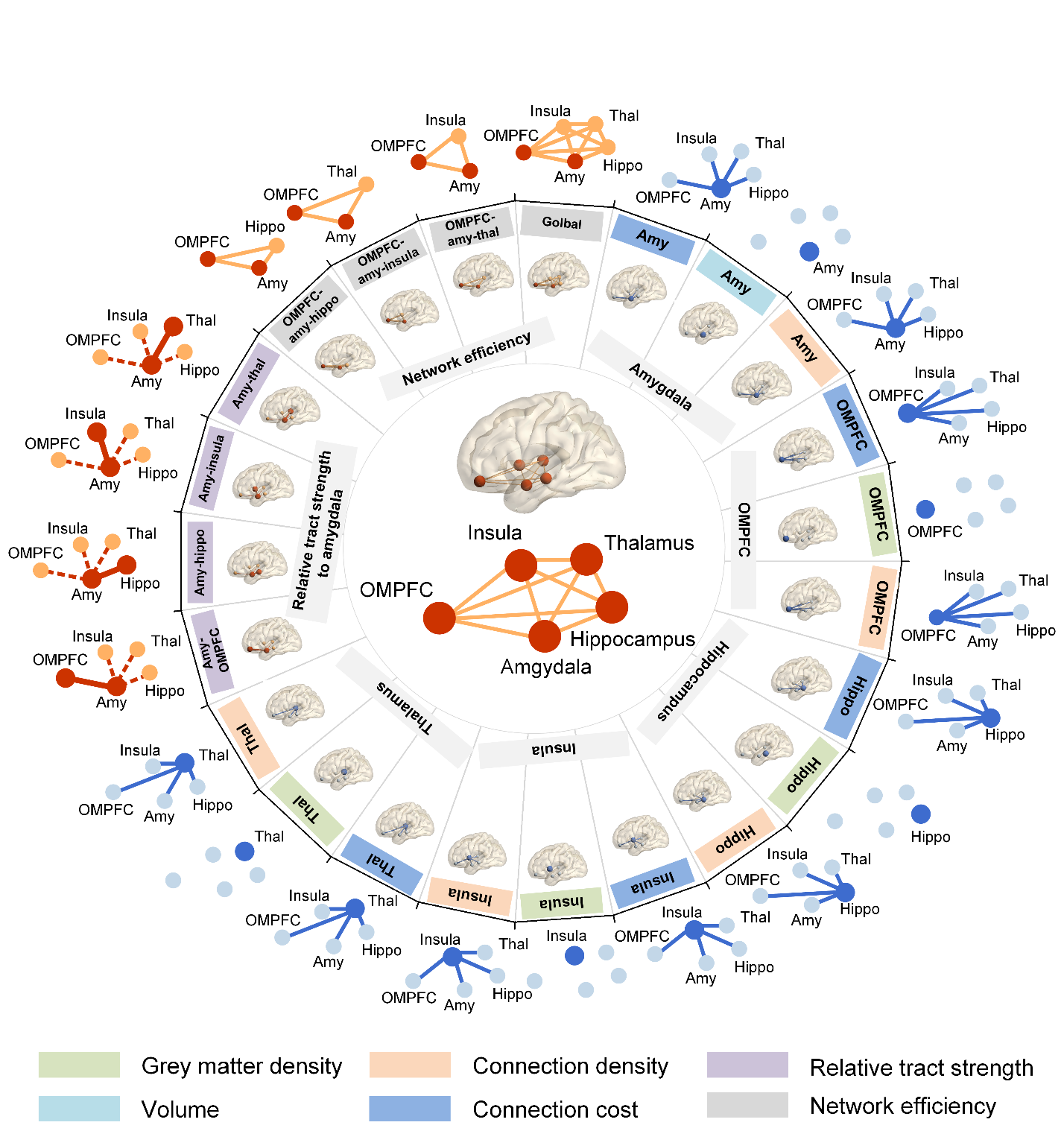
**

**Supplementary Fig.** A radar chart that displays multimodal characteristics of the amygdala, OMPFC, hippocampus, insula, and thalamus. The locations of the characteristics in this figure correspond to those in Fig 2.

**References**

1. Yoon S, Kim JE, Hwang J, Kang I, Jeon S, Im JJ, et al. Recovery from posttraumatic stress requires dynamic and sequential shifts in amygdalar connectivities. Neuropsychopharmacology. 2017;42(2):454-61. doi: 10.1038/npp.2016.136.

2. Lyoo IK, Kim JE, Yoon SJ, Hwang J, Bae S, Kim DJ. The neurobiological role of the dorsolateral prefrontal cortex in recovery from trauma: Longitudinal brain imaging study among survivors of the South Korean subway disaster. Arch Gen Psychiatry. 2011;68(7):701-13. doi: 10.1001/archgenpsychiatry.2011.70.

3. First M, Gibbon M, Spitzer RL, Williams J. User’s guide for the structured clinical interview for DSM-IV axis I disorders - research version. New York: New York State Psychiatric Institute; 1996.

4. Convit A, McHugh P, Wolf OT, De Leon MJ, Bobinski M, De Santi S, et al. MRI volume of the amygdala: A reliable method allowing separation from the hippocampal formation. Psychiatry Res Neuroimaging. 1999;90(2):113-23. doi: 10.1016/s0925-4927(99)00007-4.

5. Kim JE, Lyoo IK, Estes AM, Renshaw PF, Shaw DW, Friedman SD, et al. Laterobasal amygdalar enlargement in 6- to 7-year-old children with autism spectrum disorder. Arch Gen Psychiatry. 2010;67(11):1187-97. doi: 10.1001/archgenpsychiatry.2010.148.

6. Tzourio-Mazoyer N, Landeau B, Papathanassiou D, Crivello F, Etard O, Delcroix N, et al. Automated anatomical labeling of activations in SPM using a macroscopic anatomical parcellation of the MNI MRI single-subject brain. Neuroimage. 2002;15(1):273-89. doi: 10.1006/nimg.2001.0978.

7. Gusnard DA, Ollinger JM, Shulman GL, Cloninger CR, Price JL, Van Essen DC, et al. Persistence and brain circuitry. Proc Natl Acad Sci U S A. 2003;100(6):3479-84. doi: 10.1073/pnas.0538050100.

8. Öngür D, Price JL. The organization of networks within the orbital and medial prefrontal cortex of rats, monkeys and humans. Cereb Cortex. 2000;10(3):206-19. doi: 10.1093/cercor/10.3.206.

9. Bassett DS, Bullmore E, Verchinski BA, Mattay VS, Weinberger DR, Meyer-Lindenberg A. Hierarchical organization of human cortical networks in health and schizophrenia. J Neurosci. 2008;28(37):9239-48. doi: 10.1523/jneurosci.1929-08.2008.

10. Smith SM. Fast robust automated brain extraction. Hum Brain Mapp. 2002;17(3):143-55. doi: 10.1002/hbm.10062.

11. Zhang Y, Brady M, Smith S. Segmentation of brain MR images through a hidden Markov random field model and the expectation-maximization algorithm. IEEE Trans Med Imaging. 2001;20(1):45-57. doi: 10.1109/42.906424.

12. Zhu W, Wen W, He Y, Xia A, Anstey KJ, Sachdev P. Changing topological patterns in normal aging using large-scale structural networks. Neurobiol Aging. 2012;33(5):899-913. doi: 10.1016/j.neurobiolaging.2010.06.022.

13. Mori M, Crain BJ, Chacko VP, Van Zijl PCM. Three-dimensional tracking of axonal projections in the brain by magnetic resonance imaging. Ann Neurol. 1999;45(2):265-9. doi: 10.1002/1531-8249(199902)45:2<265::aid-ana21>3.0.co;2-3.

14. Van Den Heuvel MP, Sporns O, Collin G, Scheewe T, Mandl RCW, Cahn W, et al. Abnormal rich club organization and functional brain dynamics in schizophrenia. JAMA Psychiatry. 2013;70(8):783-92. doi: 10.1001/jamapsychiatry.2013.1328.

15. Van Den Heuvel MP, Kahn RS, Goñi J, Sporns O. High-cost, high-capacity backbone for global brain communication. Proc Natl Acad Sci U S A. 2012;109(28):11372-7. doi: 10.1073/pnas.1203593109.

16. Aron AR, Behrens TE, Smith S, Frank MJ, Poldrack RA. Triangulating a cognitive control network using diffusion-weighted Magnetic Resonance Imaging (MRI) and functional MRI. J Neurosci. 2007;27(14):3743-52. doi: 10.1523/jneurosci.0519-07.2007.

17. Cohen MX, Schoene-Bake JC, Elger CE, Weber B. Connectivity-based segregation of the human striatum predicts personality characteristics. Nat Neurosci. 2009;12(1):32-4. doi: 10.1038/nn.2228.

18. Forstmann BU, Anwander A, Schäfer A, Neumann J, Brown S, Wagenmakers EJ, et al. Cortico-striatal connections predict control over speed and accuracy in perceptual decision making. Proc Natl Acad Sci U S A. 2010;107(36):15916-20. doi: 10.1073/pnas.1004932107.

19. Latora V, Marchiori M. Efficient behavior of small-world networks. Phys Rev Lett. 2001;87(19):198701. doi: 10.1103/physrevlett.87.1987.

20. Engels JM, Diehr P. Imputation of missing longitudinal data: a comparison of methods. J Clin Epidemiol. 2003; 56(1): 968-976. doi: 10.1016/S0895-4356(03)00170-7.
